# Who are you, Griselda? A replacement name for a new genus of the Asiatic short-tailed shrews (Mammalia, Eulipotyphla, Soricidae): molecular and morphological analyses with the discussion of tribal affinities

**DOI:** 10.3897/zookeys.888.37982

**Published:** 2019-11-11

**Authors:** Anna A. Bannikova, Paulina D. Jenkins, Evgeniya N. Solovyeva, Svetlana V. Pavlova, Tatiana B. Demidova, Sergey A. Simanovsky, Boris I. Sheftel, Vladimir S. Lebedev, Yun Fang, Love Dalen, Alexei V. Abramov

**Affiliations:** 1 Department of Vertebrate Zoology, Lomonosov Moscow State University, Vorobievy Gory, 1/12, Moscow, Russia 2 The Natural History Museum, Cromwell Road, London SW7 5BD, UK Lomonosov Moscow State University Moscow Russia; 2 The Natural History Museum, Cromwell Road, London SW7 5BD, UK he Natural History Museum London United Kingdom; 3 Zoological Museum of Lomonosov Moscow State University, B. Nikitskaya 6, Moscow, Russia Zoological Museum of Lomonosov Moscow State University Moscow Russia; 4 A.N. Severtsov Institute of Ecology and Evolution, Russian Academy of Sciences, Leninskii pr. 33, Moscow, Russia A.N. Severtsov Institute of Ecology and Evolution, Russian Academy of Sciences Moscow Russia; 5 Institute of Zoology, Chinese Academy of Science, Beijing 100101, China Institute of Zoology, Chinese Academy of Science Beijing China; 6 Department of Bioinformatics and Genetics of Swedish Museum of Natural History, Stockholm, Sweden Department of Bioinformatics and Genetics of Swedish Museum of Natural History Stockholm Sweden; 7 Zoological Institute, Russian Academy of Sciences, Universitetskaya nab. 1, Saint-Petersburg 199034, Russia Zoological Institute, Russian Academy of Sciences Saint-Petersburg Russia

**Keywords:** Blarinini, Blarinellini, karyotypic variation, molecular phylogeny, *
Parablarinella
*

## Abstract

The first genetic study of the holotype of the Gansu short-tailed shrew, *Blarinella griselda* Thomas, 1912, is presented. The mitochondrial analysis demonstrated that the type specimen of *B. griselda* is close to several recently collected specimens from southern Gansu, northern Sichuan and Shaanxi, which are highly distinct from the two species of Asiatic short-tailed shrews of southern Sichuan, Yunnan, and Vietnam, *B. quadraticauda* and *B. wardi*. Our analysis of four nuclear genes supported the placement of *B. griselda* as sister to *B. quadraticauda* / *B. wardi*, with the level of divergence between these two clades corresponding to that among genera of Soricinae. A new generic name, *Parablarinella*, is proposed for the Gansu short-tailed shrew. Karyotypes of *Parablarinella griselda* (2*n* = 49, NF*a* = 50) and *B. quadraticauda* (2*n* = 49, NF*a* = 62) from southern Gansu are described. The tribal affinities of Blarinellini and Blarinini are discussed.

## Introduction

Asiatic short-tailed red-toothed shrews are commonly referred to the genus *Blarinella* Thomas, 1911. The composition of the genus *Blarinella* has been disputed for a long time. The holotype of *Sorex quadraticauda* Milne Edwards, 1872 was described from Moupin (now Baoxing) in Sichuan Province, China. Thomas (1911) considered that this species was more closely allied to the New World genus *Blarina* rather than to any Old World genus of shrews and assigned the Asian short-tailed shrews to a separate genus, *Blarinella*. In the following few years Thomas described two further species: *Blarinella griselda* Thomas, 1912 (type locality: “42 miles S.E. of Tao-chou”, = Lintan, Gansu, China) and *B. wardi* Thomas, 1915 (North Burma, “Hpimaw, Upper Burma...” = Pianma, Yunnan, China), so recognizing three species of *Blarinella*. Some subsequent authors disagreed with the species status of *griselda* and *wardi*, and regarded them either as subspecies or synonyms of *B. quadraticauda* (Allen 1938; Ellerman and Morrison-Scott 1951; Corbet 1978; Hoffmann 1987; Corbet and Hill 1992), while Hutterer (1993) considered *griselda* as a synonym of *B. quadraticauda* but *B. wardi* as a distinct species. Based on a multivariate analysis of cranial measurements, *B. griselda* and *B. wardi* were again raised to species rank (Jiang et al. 2003; Lunde et al. 2003). This treatment of *Blarinella* was accepted in MSW3 (Hutterer 2005) and other monographs (e.g. Hoffmann and Lunde 2008). The results by Jiang et al (2003) further suggested that *B. quadraticauda* is limited to west-central Sichuan and *B. wardi* is distributed from the mountains of northern Myanmar and Yunnan to the southwest of Sichuan, while *B. griselda* ranged widely from southern Gansu Province to northern Vietnam and from northwestern Yunnan to northwestern Hubei.

The analysis of one nuclear (*ApoB*) and two mitochondrial (*cytb*, *16S rRNA*) genes confirmed the distinct position of *B. wardi*, but the sequences assigned to *B. quadraticauda* (including those from the type locality in Baoxing) form a clade close to a haplogroup of the polymorphic *B. griselda* (Chen et al. 2012). Among various scenarios these authors suggested that *B. quadraticauda* is only a subspecies of *B. griselda*; however, because of the Principle of Priority within the rules of the International Commission on Zoological Nomenclature (ICZN 1999) this would be incorrect as the junior name, *B. griselda*, should be treated as a subspecies of the senior name, *B. quadraticauda*.

Recently, Bannikova et al. (2017) analyzed the genetic diversity of the genus *Blarinella* using the complete sequence of the mitochondrial gene *cytb* and four nuclear genes (*ApoB*, *BRCA2*, *RAG2*, and *IRBP*). The results of the molecular genetic analysis of samples of specimens of *Blarinella* from various locations in China and Vietnam showed that the *Blarinella* specimen from southern Gansu stands apart from the other representatives of the genus and could not be assigned to a known species based on the molecular data available at that time. This individual (ZMMU S-195179, ID Chi111) was karyotyped and its chromosome set (2*n* = 49, NF*a* = 50) was described in Sheftel et al. (2018) without an illustration. Previously only two karyotypes of Asiatic short-tailed shrews have been described: *B. wardi* (2*n* = 32, NF*a* = 58) from Yunnan Province (Moribe et al. 2007) and *B.* “*griselda*”(2*n* = 44) (Ye et al. 2006) also from Yunnan Province, Nanjian County (Dr Chen Zhongzheng pers. comm. 2016).

The distinct position of the Gansu specimen led us to continue with further studies to re-evaluate the taxonomic status of the Asiatic short-tailed shrews from China and Vietnam. In the meantime, a new generic name was proposed based on the previously published data of our specimen from Gansu and additional specimens from Shaanxi (He et al. 2018). The new genus was named as *Pantherina* He, 2018 with *Blarinella griselda* Thomas, 1912 as the type species. Unfortunately, the authors made a nomenclatural error, since the name *Pantherina* He, 2018 is preoccupied by *Pantherina* Curletti, 1998, which was proposed as a subgeneric name for the African beetles (Coleoptera, Buprestidae, *Agrilus*) (see Curletti 1998). According to the ICZN (1999) a new name should be proposed for the Asiatic short-tailed shrew from Gansu.

Another question arises over the attribution of the new name to *B. griselda* Thomas, 1912 from Gansu, because no direct comparison with the type specimen was made by He et al. (2018). None of the previous studies (Jiang et al. 2003; Chen et al. 2012; He et al. 2018) analyze the holotype of *B. griselda*. The only shrew from Gansu included in the molecular analysis by He et al. (2018) was represented by previously published *cytb* sequence of the specimen ZMMU S-195179 from Bannikova et al. (2017). The only shrew from Gansu included in their craniometric analysis was specimen AMNH M-60499 (labeled as “Kansu”) using data retrieved from the paper of Jiang et al. (2003). However, this specimen was never sequenced and the skull of this specimen from the American Museum of Natural History (AMNH) was lost a long time ago (Dr Ross MacPhee pers.comm. April 2016). According to their craniometric data this specimen falls into the same morphological cluster together with *B. quadraticauda* and “*Pantherina*” specimens from Shaanxi Province. Therefore, as neither the holotype specimen nor topotypes of *B. griselda* were included by He et al. (2018) for the morphological diagnosis of the newly described genus, their diagnosis was based on the specimens from Shaanxi Province.

This current study presents the first molecular study of the holotype of *B. griselda* and includes new data on additional specimens of this rare species from Gansu and northern Sichuan. The new name for this taxon is provided below.

Allen (1938) disagreed with Thomas (1911) about the supposed relationship of *Blarinella* with *Blarina* and, based on external, cranial and dental morphology, was of the opinion that *Blarinella* was more closely related to *Sorex*. Repenning (1967) shared this view and in his division of the Soricinae into three tribes, he placed *Blarinella* in the tribe Soricini, with *Blarina* and *Cryptotis* in the tribe Blarinini. Reumer (1998) considered that *Blarinella* and eight related fossil genera should be separated from the Soricini and placed in a new tribe, the Blarinellini. This useful tribal arrangement based entirely on morphology has been accepted and widely followed; however, some of the results of recent molecular studies (Dubey et al. 2007) have suggested that the tribal arrangement may not be so well defined and is in need of revision. Thus, on the basis of our new data, another task was to revise the arrangement of the Blarinini/Blarinellini tribes.

## Material and methods

### Taxon sampling and tissue collection

The specimens of Asiatic short-tailed shrews were collected during the surveys of small mammals conducted by the Russian Academy of Sciences and the Chinese Academy of Sciences in Gansu and Sichuan provinces of China. Voucher specimens are deposited in the Zoological Museum of Lomonosov Moscow State University (**ZMMU**). These specimens were compared with the Asiatic short-tailed shrews kept in the collection of the Natural History Museum, London, UK (**NHMUK**) and the Zoological Institute of the Russian Academy of Sciences, Saint Petersburg, Russia (**ZIN**). Among them, the holotype of *B. griselda* (NHMUK 1912.8.5.23) was genetically studied for the first time. On the whole, we obtained 23 new sequences from 11 specimens of *Blarinella* and two specimens of *Chodsigoa hypsibia* (Table 1). For the phylogenetic analysis, 47 mitochondrial and nuclear sequences of *Blarinella*, *Blarina*, *Chodsigoa*, *Chimarrogale*, *Neomys*, *Anourosorex*, and *Sorex* from our previous studies (Abramov et al. 2017b; Bannikova and Lebedev 2010; Bannikova et al. 2015, 2017, 2018) and additional 116 sequences of different genera of Soricinae as well as Crocidurina (*Crocidura fuliginosa*) from GenBank were used (see Suppl. material 1: Table S1).

**Table 1.** List of the original material used in the molecular study and specimens examined in the morphological analysis: species, specimen ID, collection and geographic origin. Samples are stored in the following collections: ZMMU – Zoological Museum of Moscow State University, Russia; ZIN – Zoological Institute of Russian Academy of Sciences, St.-Petersburg, Russia; NHMUK – Natural History Museum, London, UK. All specimens in the phylogenetic analysis were also included in the morphological analysis, with the exception of those marked thus – #.

**Species**	**Specimen code in phylogenetic analysis (Figs 2, 3, Suppl. material 1: Figure S1)**	**Museum catalogue number and/or field collection code (in brackets)**	**Collecting locality (country, province and closest city)**
“*Blarinella*” *griselda*	NHMUK	NHMUK 1912.8.5.23 Holotype	China, Gansu, 68 km SE Taochou (Lintan), Tsingling (Qinling) Mountains, 34°40’N, 103°35’E
	Chi111	ZMMU S-195179	China, S. Gansu, Taizishan NR, 35°16’N, 103°26’E
		ZMMU S-199245	China, S. Gansu, Taizishan NR, 35°16’N, 103°26’E
	G17-87	ZMMU G17-87	China, N. Sichuan, Ruoergai (Zoige), 33°35’N, 103°09’E
	G18-252	ZMMU G18-252	China, N. Sichuan, Songpan, 32°30’N 103°35’E
*B. quadraticauda*	Bl-1	ZIN 91211 (36) #	Vietnam, Lao Cai, Van Ban, 21°58’N, 104°02’E
	Bl-2	ZIN 96272 (42) #	Vietnam, Lao Cai, Sa Pa, 22°21’N, 103°46’E
	Bl-3	ZIN 96273 (43) #	Vietnam, Lao Cai, Sa Pa, 22°21’N, 103°46’E
		ZIN 98268	Vietnam, Lao Cai, Sa Pa, 22°21’N, 103°46’E
		ZIN 99935	Vietnam, Lao Cai, Sa Pa, 22°21’N, 103°46’E
	Bl-5	ZIN 97788 (136)	Vietnam, Lao Cai, Sa Pa, 22°21’N, 103°46’E
	V12-40	ZIN 101574	Vietnam, Lao Cai, Sa Pa, 22°21’N, 103°46’E
	V12-61	ZIN 101575	Vietnam, Lao Cai, Sa Pa, 22°21’N, 103°46’E
B. cf. quadraticauda	G17-12	ZMMU G17-12	China, S. Gansu, Huixian, 33°40’N 106°15’E
*B. quadraticauda*		NHMUK 1911.9.8.56	China, S. Sichuan, Omi-San (Emei Shan), 29°30’N, 103°18’E
		NHMUK 1911.9.8.57	China, S. Sichuan, Omi-San (Emei Shan), 29°30’N, 103°18’E
		NHMUK 1911.9.8.58	China, S. Sichuan, Omi-San (Emei Shan), 29°30’N, 103°18’E
		NHMUK 1911.9.8.59	China, S. Sichuan, Omi-San (Emei Shan), 29°30’N, 103°18’E
		NHMUK 1911.9.8.25	China, S. Sichuan, Nan-chwan (Nanchuan), 29°07’N, 107°16’E
*B. wardi*		NHMUK 1915.2.1. Holotype	China, Yunnan, Hpimaw, {formerly Upper Burma / Myanmar} (Pianma), 26°N, 98°35’E (26°00’N, 98°37’E)
		NHMUK 1932.11.1.33	Myanmar, Adung Valley, 28°15’N, 97°40’E
		NHMUK 1922.9.1.26	China, Yunnan, Mekong – Salwin (Salween) Divide, 28°N (c. 27°30’N 98°56’E / 28°20’N 98°44’E)
		NHMUK 1922.9.1.27	China, Yunnan, Kiu-Kiang – Salwin Divide, 28°N (c. 28°40’N 98°15’E)
*Chodsigoa hypsibia*	Chi11-72	ZMMU S-195190	China, S. Gansu, Lianhuashan NR, 34°56’N, 103°44’E
	G17-13	ZMMU G17-13	China, S. Gansu, Huixian, 33°40’N 106°15’E

**Figure 1.**
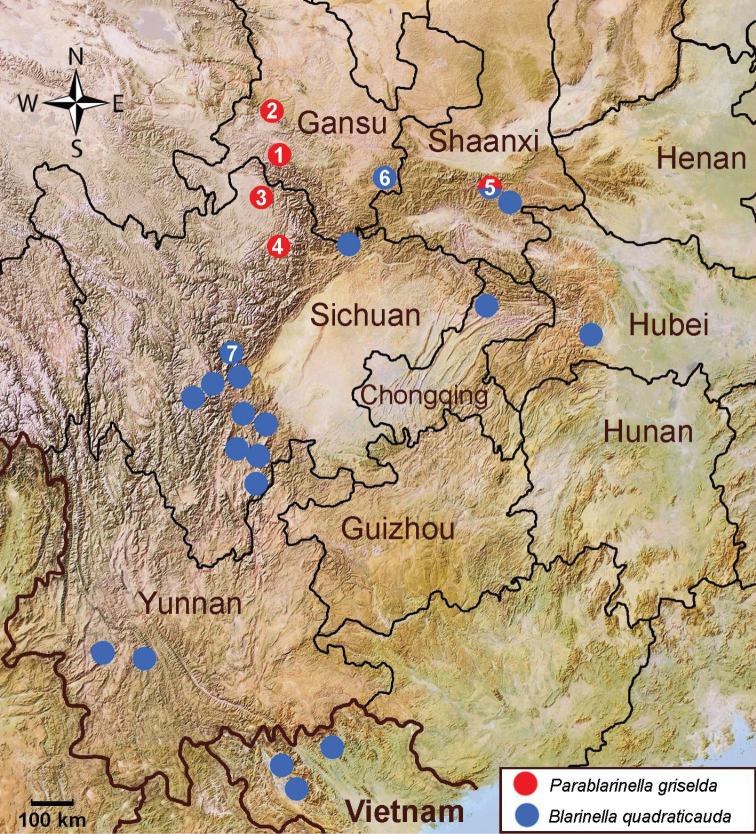
Sample localities of specimens used for molecular analyses **1** China, Gansu, Lingtan County (NHMUK 12.8.5.23, holotype of *Blarinella griselda* Thomas, 1912) **2** China, Gansu, Taizishan NR (ZMMU S-195179) **3** China, Sichuan, Ruoergai (Zoige) (ZMMU G17-87) **4** China, Sichuan, Songpan (ZMMU G18-252) **5** China, Shaanxi, Mt.Qinling (after He et al. 2018) **6** China, Gansu, Huixian (ZMMU G17-12) **7** China, Sichuan, Baoxing (type locality of *Blarinella quadraticauda* (Milne-Edwards, 1872)). Unnumbered localities based on the GenBank data.

### DNA extraction, PCR amplification, and sequencing

Genomic DNA from ethanol-preserved tissues of the recent specimens was extracted using a standard protocol of proteinase K digestion, phenol-chloroform deproteinisation and isopropanol precipitation (Sambrook et al. 1989). We sequenced the complete mitochondrial cytochrome *b* (*cytb*) gene and fragments of four nuclear loci: apolipoprotein B (*ApoB*), exon 11 of the breast cancer type 1 susceptibility protein (*BRCA1*), recombination activating gene 2 (*RAG2*), and the interphotoreceptor retinoid binding photoreceptor (*IRBP*). Primers and polymerase chain reaction protocols for nuclear loci (*ApoB*, *BRCA1*, *IRBP* and *RAG2*) are described in Abramov et al. (2017a) and Bannikova et al. (2018). New primers were designed specifically for amplification and sequencing the complete *cytb* gene and its short fragments from the historical museum specimen, the holotype of *B. griselda* (Suppl. material 1: Table S2). General methods for the amplification and sequencing of *cytb* from recent samples are described in Bannikova et al. (2010). PCR products were sequenced on the autosequencing system ABI 3100-Avant using ABI PRISM BigDyeTM Terminator v. 3.1 (Applied Biosystems, Foster City, CA, USA).

### Molecular analysis of the holotype of *B. griselda*

Molecular analysis (DNA extraction and PCR preparation) of the holotype of *B. griselda* NHMUK1912.8.5.23 was performed in the laboratories of Department of Bioinformatics and Genetics of the Swedish Museum of Natural History in the special laboratory for historical museum samples. DNA was extracted from a ~1.5 mm × 1.5 mm skin sample that was washed in ethanol prior to initiating the extraction procedure.

Extraction of DNA was performed using Qiagen QIAamp DNA Micro Kit following the protocol “Isolation of Genomic DNA from Tissues” with some changes: (1) additional 5 µl of Proteinase K after overnight lysis and incubation at room temperature for 30 min; (2) two steps of elution with AE buffer, each with 20 µl of buffer and 5 min of incubation at room temperature. Amplification of *cytb* fragments was performed in 25 µl reaction volume containing 1–2 µl DNA, 2.5 µl 10× buffer, 1 µl of each primer (10 pmol/µl), 0.5 µl of dNTP mix, 1 µl MgCl2, 2.5 µl BSA, and 0.4 µl Taq polymerase. Extraction was performed twice, the second time with blank as a negative control. Double-stranded polymerase chain reaction entailed 50-thermal cycles and was performed as follows: 95 °C for 10 min, (94 °C for 30 sec, 52 °C for 30 sec, 72 °C for 30 sec) ×50 cycles, 72 °C for 7 min, 12 °C for forever. Negative controls were used both for DNA and PCR mix. PCR products were verified on 1% agarose gels stained with Gel Green. Primer pairs which resulted in bands on the gel and empty negative controls: L467x – H601x, L240a – H400a, L62x – H190x, L170ax – H330x, L580x – H670x. Amplicons were sequenced directly by Sanger sequencing on Applied Biosystems 3130xl Genetic Analyzer. Each fragment was sequenced several times to ensure the authenticity of the sequence.

It is known that DNA undergoes degradation over time (Hofreiter et al. 2001), such that mtDNA sequences from even relatively recent museum specimens can exhibit sequencing artifacts (Sefc et al. 2007). Most errors involves C→T changes, presumably due to the deamination of cytosine bases in the template (Hofreiter et al. 2001). Here, neither clear double C/T peaks nor an excess of C→T transitions were observed in the *cytb* sequences obtained from the historical *Blarinella* sample relative to modern samples, suggesting that the holotype sequences are authentic.

The sequences obtained in this study can be accessed via GenBank (accession numbers: MN199101 to MN199123, Suppl. material 1: Table S1).

### Alignment, partitioning, and phylogenetic tree reconstruction

All sequences were aligned by eye using Bioedit v. 7.0.9.0 (Hall 1999). Heterozygous positions in nuclear genes were coded using the IUB ambiguity codes and sequences were used as unphased genotypes. The ModelFinder routine (Kalyaanamoorthy et al. 2017) as implemented in IQTREE v. 1.6 (Nguyen et al. 2015) was used to determine the optimum partitioning scheme and the best-fit substitution models for each subset under the BIC criterion.

Phylogenetic reconstructions were performed with each nuclear gene separately and all nuclear genes combined. Phylogenetic trees were reconstructed from nuclear concatenation under Maximum Likelihood (ML) and Bayesian criteria. Maximum likelihood reconstructions were conducted in IQTREE v. 1.6 (Nguyen et al. 2015). Clade stability was tested using Ultrafast Bootstrap (Minh et al. 2013) with 10 000 replicates. Bayesian tree reconstructions were performed in MrBayes v. 3.2 (Ronquist et al. 2012). Models with either two or six rate matrix parameters were selected for each subset using ModelFinder. For most parameters, default priors were used. Compound Dirichlet priors for branch lengths combined with gamma prior on the tree length were invoked. All parameters except branch lengths were unlinked across partitions. The analysis included two independent runs of four chains with the default heating scheme. The chain length was set at 20 million generations with the sampling of every 10 000 generation. Tracer v. 1.6 software (Rambaut and Drummond 2003) was used to check for convergence and to determine the necessary burn-in fraction, which was 10% of the chain length. The effective sample size exceeded 200 for all estimated parameters.

The mitochondrial phylogeny of *Blarinella* was generated from the *cytb* alignment containing 46 sequences of Asiatic short-tailed shrews including the partial sequence of the holotype of *Blarinella griselda*. The ML and Bayesian trees were reconstructed as described above and rooted using *Blarina* and *Cryptotis* as the outgroups. In addition, we performed the analyses of the extended set of taxa of Soricinae with the aim to examine more thoroughly the relationships among taxa of the Blarinini-Blarinellini clade and other soricine genera. The *p*-distances were calculated in PAUP* v. 4.0b10 (Swofford 2003).

## Molecular dating

Molecular dating was performed in BEAST v. 1.84 based on the nuclear dataset. The optimum partitioning scheme and substitution model were determined separately for each gene in ModelFinder (Kalyaanamoorthy et al. 2017). Based on the results of the hierarchical likelihood ratio tests performed in PAUP* v. 4.0b10 (Swofford 2003) strict clock models were employed. The analysis was conducted using birth-death tree shape prior. The chain length was set at 100 million generations, the effective sample size exceeded 200 for all estimated parameters after the 10% burn-in fraction was discarded. The tree was calibrated using a secondary calibration point corresponding to the time of divergence of Blarinini and Blarinellini (normal distribution with 15.38 million years (My) as the mean and 2.34 My as the standard deviations). In addition, an informative prior for the *ApoB* clock rate was employed (lognormal distribution with 2.42 E^–3^ as the mean and 2.59 E^–4^ as the standard deviations).The parameters of these prior densities are equivalent to those of the posterior distribution produced by the Bayesian molecular clock analysis of the multilocus data on Soricinae (Bannikova et al. 2018). Taking into account potential ambiguity in interpretation of fossil data on Blarinini and Blarinellini (Doby 2015) the dataset of Bannikova et al. (2018) was re-analyzed omitting the calibration concerning the latter two taxa.

## Morphology

Specimens sampled for the phylogenetic analysis and included in the morphological analysis were compared with historical material of all three taxa in the NHMUK collection (Table 1). For the historical material, place names and their coordinates were determined from field notes in combination with information obtained from the United States Board on Geographic Names (**USBGN**), the GEOnet Names Server (**GNS**) (http://geonames.nga.mil/gns/html/) and Google Earth (http://earth.google.com).

External measurements of historical specimens are those recorded by collectors on specimen labels. Recently collected specimens and the crania of all specimens were measured in millimetres using digital callipers. Cranial and dental nomenclature follows that of McDowell (1958), Meester (1963), Mills (1966), Repenning (1967), Butler and Greenwood (1979), Reumer (1984), and Dannelid (1998). Abbreviations used in the text for the dental nomenclature are incisor (I/i), unicuspid (Un), lower antemolar (a), premolar (P/p), and molar (M/m), with premaxillary and maxillary teeth denoted by uppercase and mandibular teeth by lowercase letters.

### Karyotyping

Karyotypes of two Asiatic short-tailed shrews, a male ZMMU S-195179 (ID Chi111) from southern Gansu, Taizishan and a female G17-12 from southern Gansu, Huixian were examined. Mitotic chromosome preparations were made in the field from both bone marrow and spleen after colchicine treatment *in vivo* following Ford and Hamerton (1956) with some modifications (Bulatova et al. 2009).

In case of the male, preparations were made from spleen using a simple technique without centrifugation proposed by Krysanov et al. (2009). Briefly, after colchicine treatment *in vivo* an incised spleen incubates with 5 mL of KCl hypotonic solution (0.07 M) for 20–30 min at room temperature, and fix with 5 mL of freshly prepared glacial acetic-methanol (1:3) for 5 min twice. Such samples can be stored at −10 °C up to 6 months. To prepare air-dried slides a fixed tissue incubates with 200 µl of 50% glacial acetic acid for 3–4 min, resuspends, and then drops suspension onto a hot slide (30 min at 90 °C). After drying, a slide incubates with pure methanol for 5 min, and then dried again. Air-dried chromosome spreads of both specimens were stained conventionally with 4% Giemsa for 8 min.

CBG-banding was performed using the standard technique (Sumner 1972) to determine C-heterochromatin blocks (for the female karyotype only).

## Results

### Alignment and partitioning

The total matrix used in the *cytb* analysis (74 sequences, 1140 bp) contained 46 specimens of *Blarinella* and 28 specimens of other soricids. Models for the *cytb* gene estimated by IQTREE and employed in the Maximum likelihood analysis were as follows: 1^st^ codon position TIM2e+I+G4, 2^nd^ codon position TIM3+F+I+G4, and 3^rd^ codon position TIM2+F+I+G4.

In the combined analyses of four nuclear genes, the final alignment consisted of 2914 nucleotide positions, including 472 bp of *ApoB*, 840 bp of *BRCA1*, 741 bp of *RAG2*, and 861 bp of *IRBP*. In total, the nuclear dataset contained 34 specimens, including 19 outgroups. We also performed a separate analysis of the extended *ApoB* data (44 sequences, 472 bp) because this nuclear gene is represented by the largest number of *Blarinella* sequences in GenBank. The best-fit substitution models employed for each of the five partitions found by IQTREE are given in Suppl. material 1: Table S3.

### Position of the holotype of *B. griselda* on the mitochondrial cytb tree

**Figure 2.**
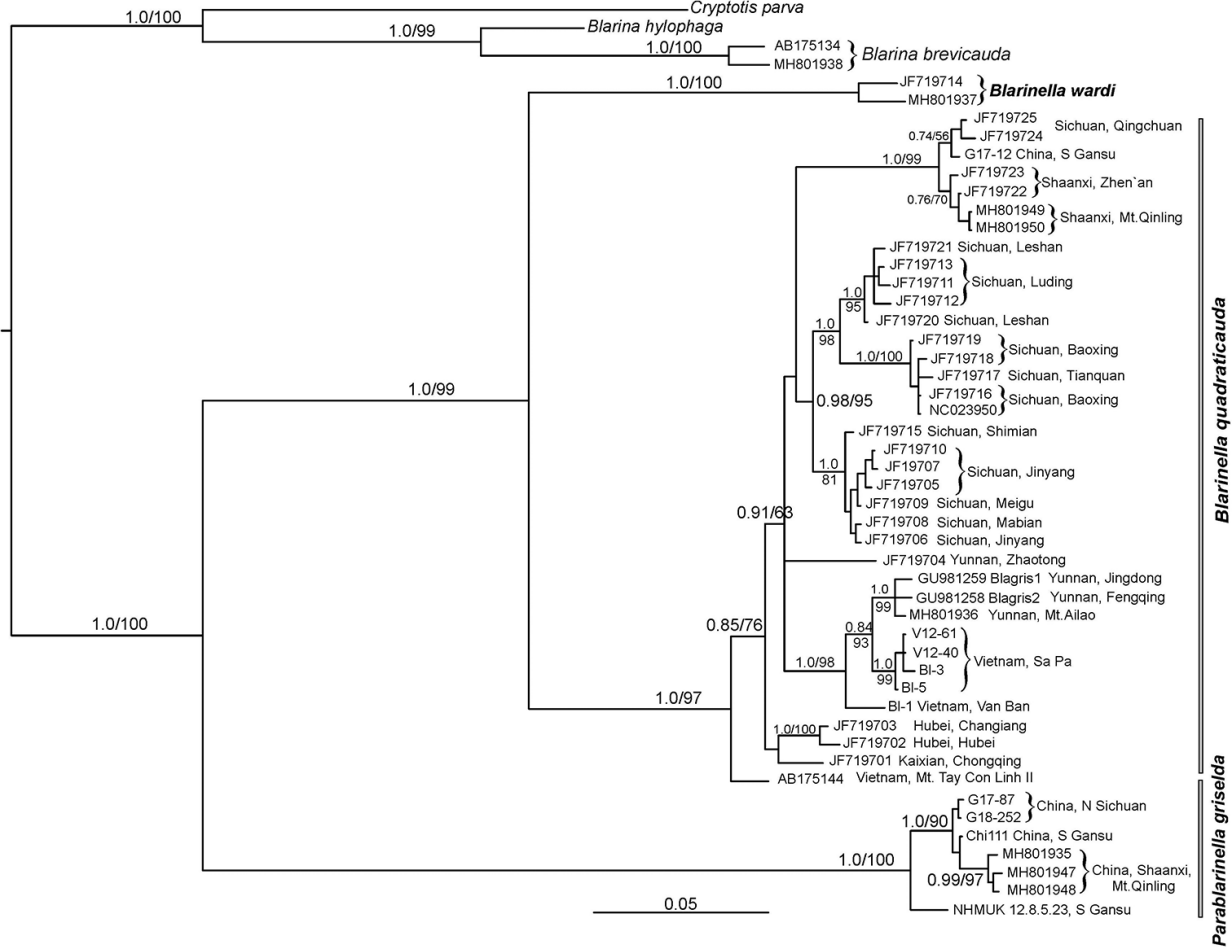
The phylogenetic relationships in *Blarinella* as reconstructed in MrBayes based on *cytb* data. Numbers above or below branches correspond to Bayesian posterior probabilities and ML bootstrap values (>50%) generated using fast bootstrap algorithm in IQTREE. The genera *Blarina* and *Cryptotis* are used as outgroups.

As a result of the genetic analysis of the type specimen of *B. griselda*, we obtained sequences of three fragments of *cytb*: 90, 160 and 120 bp. The analysis of these fragments showed that the holotype of *griselda* is very close to our specimen from southern Gansu, two other specimens from northern Sichuan and three specimens from Shaanxi named as *Pantherina* in He et al. (2018) (Fig. 2; Suppl. material 1: Fig. S1). Together all seven specimens form a clade which appears distinctly separate from all the species of *Blarinella* (*p*-distance ~19%).

Overall, three clades of the species of *Blarinella* may be recognized in the *cytb* tree: (I) the first clade consists of *B. wardi* (described from Myanmar and found also in western Yunnan); (II) the second one corresponds to *B. quadraticauda*: these are specimens previously identified as *B. griselda* from different localities in China and northern Vietnam and *B. quadraticauda* from Sichuan Province, including specimens from Baoxing (the type locality of *B. quadraticauda*); this clade stands as a sister branch to *B. wardi*; (III) the third clade contains the holotype of *griselda*, one specimen from southern Gansu, two specimens from northern Sichuan and three specimens from Shaanxi; it is highly divergent from clades I and II. Based on these data combined with morphological and nuclear results presented below, we consider this third clade rather as a separate genus, hereinafter referred to as *Parablarinella*. A detailed justification of this decision and the description of the new taxon is given in the Discussion.

### Phylogenetic analysis of the species based on nuclear genes and molecular time estimation

**Figure 3.**
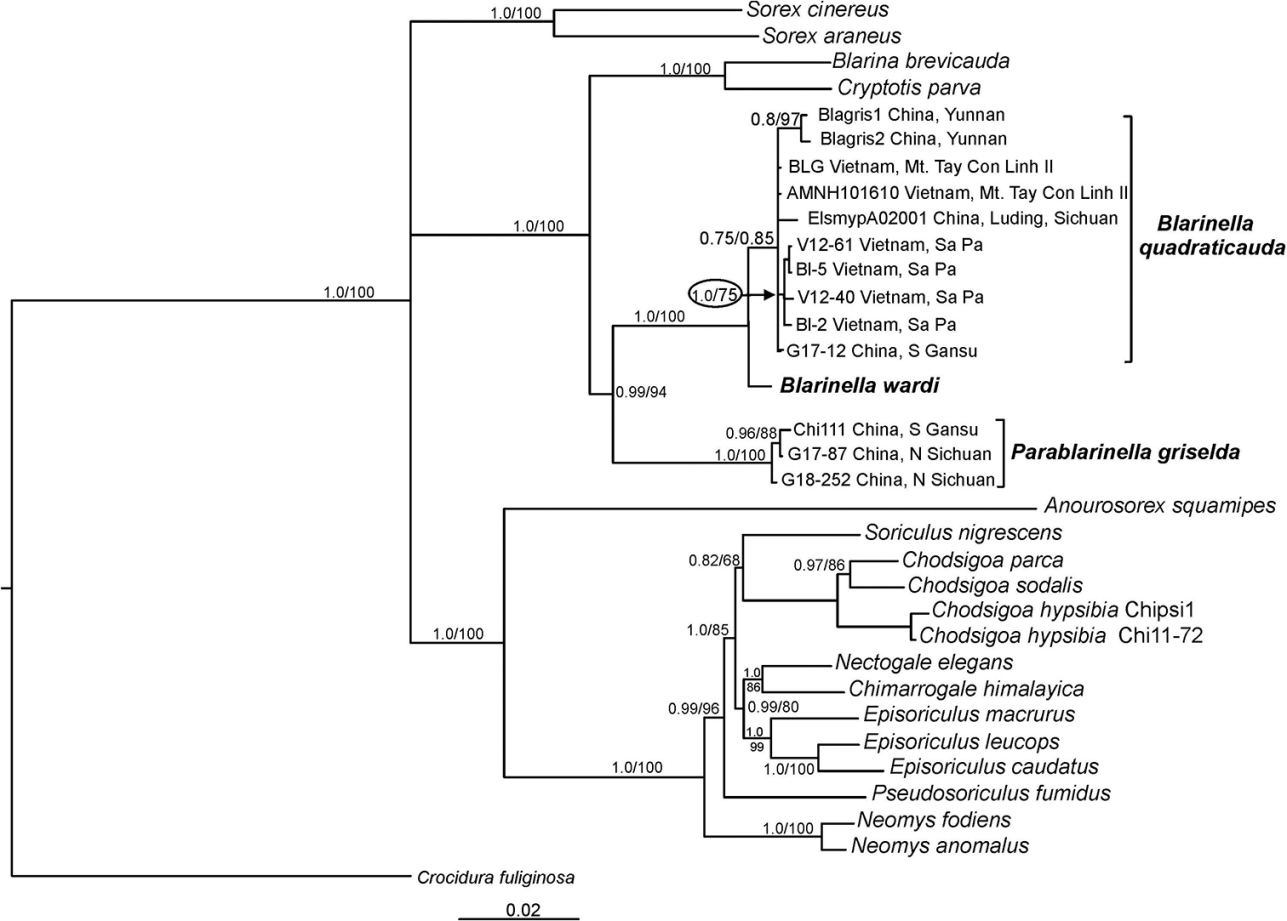
MrBayes tree of Soricinae genera as inferred from the concatenation of four nuclear genes. Numbers above or below branches correspond to Bayesian posterior probabilities and ML bootstrap values (>50%) generated using fast bootstrap algorithm in IQTREE. *Crocidura fuliginosa* is used as outgroup.

Phylogenetic analysis of the relationships of the species of *Blarinella* based on nuclear genes (Fig. 3; Suppl. material 1: Fig. S2) supported the separate position of the specimens that were close to the holotype of *B. griselda* on the mitochondrial tree (Fig. 2). This clade occupied the sister position to all the remaining species of *Blarinella* (0.99/94). This pattern is consistent with the previous results by Bannikova et al. (2017) and He et al. (2018). The genetic distance between the *B. griselda* clade (*Parablarinella*) and the *B. quadraticauda* clade is higher than that between the genera *Blarina* and *Cryptotis* (*p*-distance ~0.5% and 0.038% accordingly). The specimens of *B. quadraticauda* formed a single clade sister to *B. wardi* (1.0/100).

The relatively close relationship of the *Blarina* / *Cryptotis* clade to the *Blarinella* / *Parablarinella* clade is clearly shown in Figure 3, demonstrating that these taxa form a clade separate from the other soricid outgroups. Divergence times as inferred from nuclear concatenated data using BEAST (Table 2) are nearly half of those obtained in He et al. (2018) based on mitogenomic data, which is likely explained by a bias due to saturation in the fast evolving mitochondrial DNA. The estimated divergence time between *Parablarinella griselda* and *Blarinella* proper is 9.61 My (95% HPD = 6.87–12.74), that is ~1.5 times higher than the most recent common ancestor (tmrca) of Blarinini. The tmrca of *B. quadraticauda* and *B. wardi* was estimated at 1.68 My (95% HPD = 0.71–3.25).

**Table 2.** Approximate node age estimates (My) in Blarinellini based on nuclear data.

**Node of species, clades or subclades**	**Age (My)**	**95% HPD**
Blarinini/Blarinellini	12.39	9.29–15.93
Tmrca Blarinini (*Blarina*/*Cryptotis*)	5.99	3.81–8.12
Tmrca Blarinellini (*Parablarinella/Blarinella*)	9.61	6.87–12.74
Tmrca *Blarinella* (*B. wardi*/*B. quadraticauda*)	1.68	0.71–3.25
Tmrca *B. quadraticauda*	1.11	0.71–3.25
Tmrca *Parablarinella griselda*	0.45	0.16–0.89

### Morphology

The three species are very similar in external appearance; the tail is approximately half the length of the head and body (48–63% in *B. quadraticauda*, 50–61% in *B. wardi* and 47–51% in *B. griselda*). The eyes are small, the ears small and almost completely concealed in the pelage, the claws on all feet are moderately large, and a gland is indicated on the mid-ventral surface of males.

**Figure 4.**
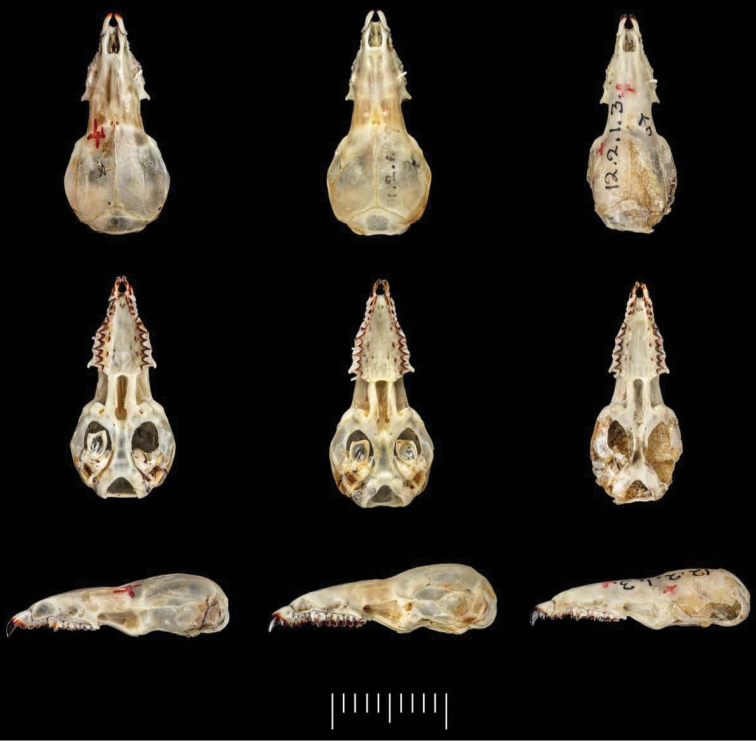
Skulls from left to right of the holotype of *Parablarinella griselda*NHMUK 1912.8.5.23; *Blarinella quadraticauda*NHMUK 1911.2.1.59; the holotype of *Blarinella wardi*NHMUK 1915.2.1.3 (please note that the number written incorrectly as 12.2.1.3 on the skull of this species should read 15.2.1.3). Top row: dorsal view; middle row: ventral view; lower row: left lateral view.

**Figure 5.**
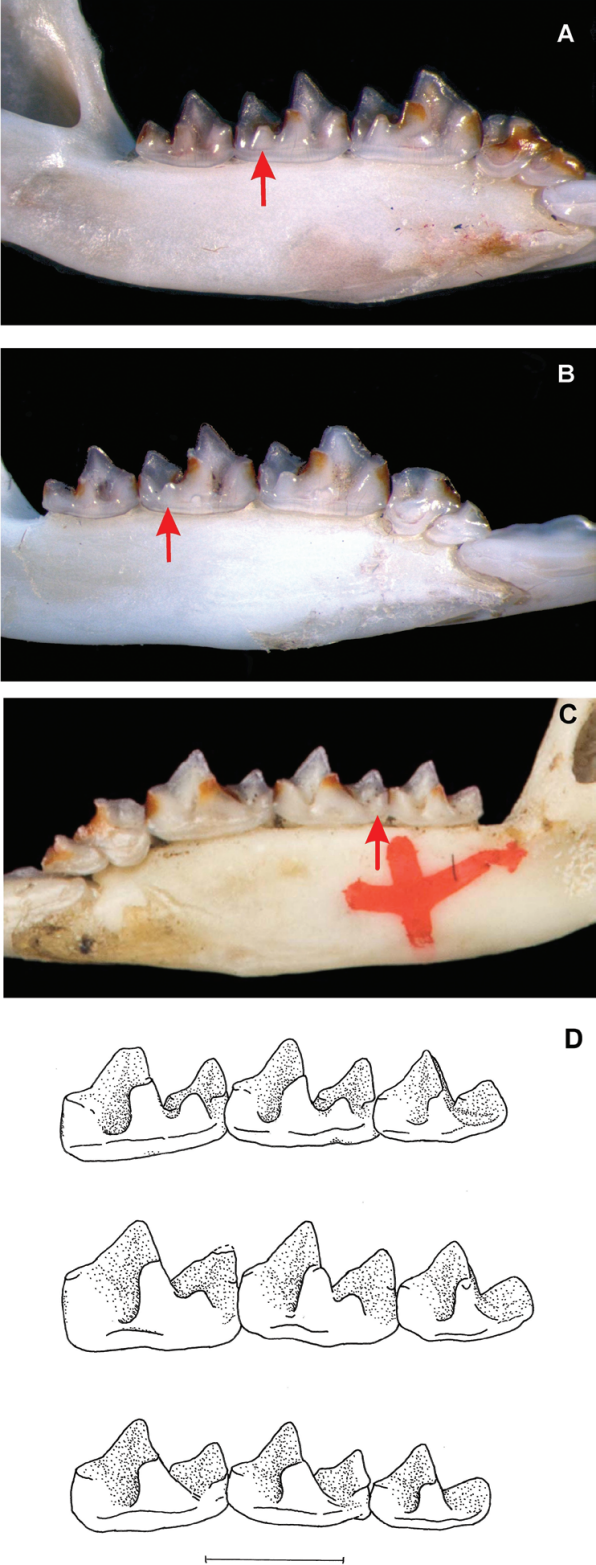
Variation in morphology of the talonid of the lower molars **A** lingual view of left mandibular ramus of *Parablarinella griselda*ZMMU G18-252 **B** lingual view of left mandibular ramus of *Blarinella quadraticauda*ZMMU G17-12 **C** lingual view of right mandibular ramus of holotype of *Blarinella wardi*NHMUK 1915.2.1.3 **D** comparison of right lower molars to show variation in development of the entoconid and entoconid crest on m1 and m2 and the talonid of m3. Above holotype of *Parablarinella griselda*NHMUK 1912.8.5.23, middle *Blarinella quadraticauda*NHMUK 1911.2.1.57, below holotype of *Blarinella wardi*NHMUK 1915.2.1.3. The arrows indicate the entoconid and entoconid crest on m2. Scale bar: 1 mm.

**Figure 6.**
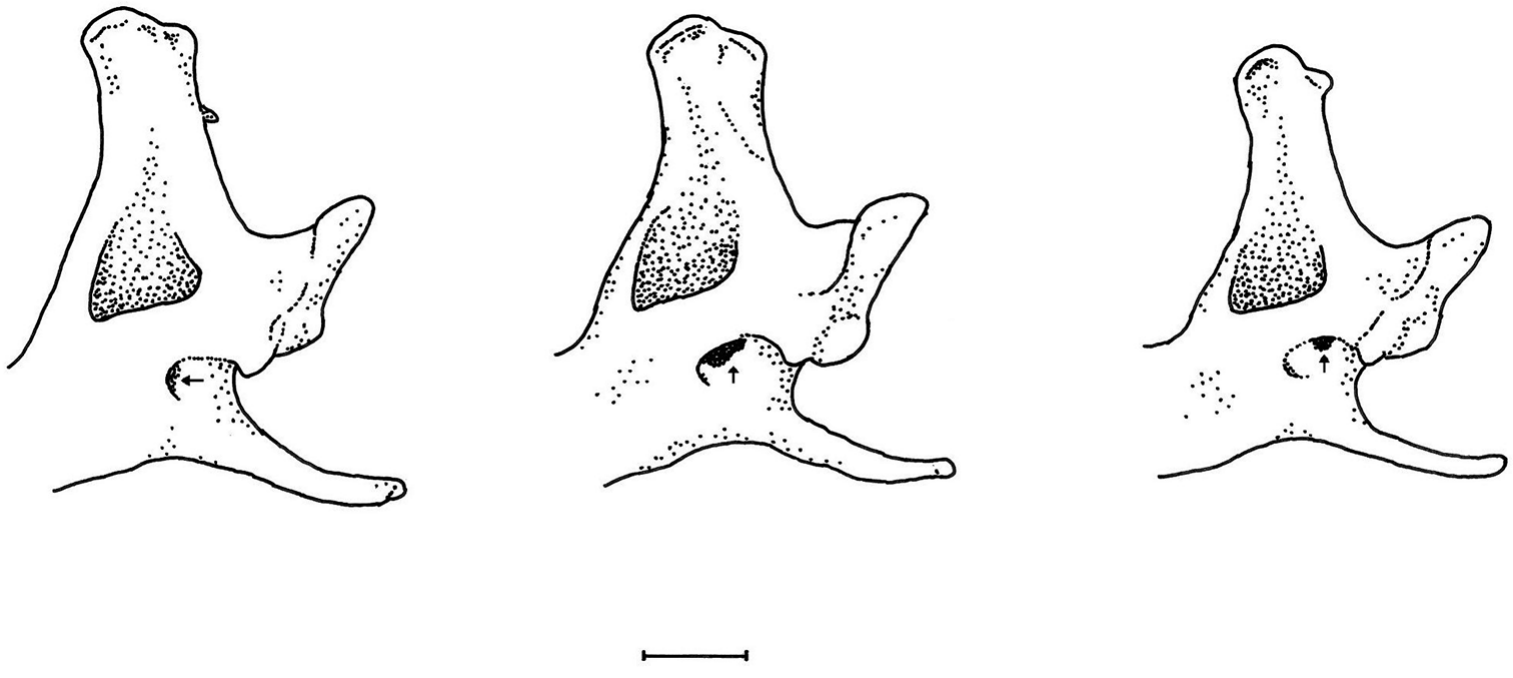
Comparison of lingual view of posterior region of right mandible to show variation in mandibular and ramal foramina. Mandibular foramen: horizontal arrow; ramal foramen: vertical arrow. From left to right: holotype of *Parablarinella griselda*NHMUK 1912.8.5.23; *Blarinella quadraticauda*NHMUK 1911.2.1.59; holotype of *Blarinella wardi*NHMUK 1915.2.3. Scale bar: 1 mm.

Despite the marked genetic divergence between “*B. griselda*” (*Parablarinella*) and the other two species, differences in cranial and dental morphology are comparatively limited and not as great as might be expected to distinguish genera, and for some characters there is equal variation between *B. quadraticauda* and *B. wardi* as between either one of these species and *Parablarinella*. In their description of the new genus, He et al. (2018) presented characters to separate *B. griselda* from the other two species. Here, based on historical material and the recently acquired specimens available to us, we elaborate on these characters and provide additional ones. The craniodental characters that in combination serve to distinguish the three taxa, and those that separate the two genera, are shown in Table 3 and Figures 4–6.

**Table 3.** Comparison of dental and cranial morphology of *Blarinella quadraticauda*, *B. wardi* and *Parablarinella griselda*.

**Character**	***Parablarinella griselda***	***Blarinella quadraticauda***	***Blarinella wardi***
I1 angle of principal to posterior cusp	Moderately shallow > 90°	Moderately acute, approaching or < 90°	Moderately acute
Relative size of unicuspids (Fig. 4)	Un1>Un2>Un3>>Un4>Un5	Un1>Un2>>Un3>>Un4>Un5 or Un1>Un2>>Un3>>Un4>>Un5	Un1>Un2>>Un3>>Un4>Un5 or Un1>Un2>>Un3>>Un4sub=Un
Size of Un3	Un3 smaller than Un2	Un3 markedly smaller than Un2	Un3 markedly smaller than Un2
	Height c. 0.6 – 0.75, volume c 0.7 – 0.75 of Un2	Height c 0.45 – 0.6, volume c 0.5 – 0.6 of Un2	Height 0.5 – 0.6, volume 0.5 – 0.6 of Un2
Size of Un4	Height c 0.4 – 0.45, volume c 0.33 – 0.6 of Un3	Height c 0.5 – 0.75, volume c 0.5 – 0.75of Un3.	Height c 0.6, volume c 0.5 – 0.75 of Un3.
P4 shape	Lingual margin of tooth curved. Ratio of anterior to posterior width moderate, tooth noticeably broader posteriorly than anteriorly. Hypocone absent; narrow trough between anterior of cingulum and protocone. Lingual cingulum forms a shallow semi-circle; postero-lingual margin projects beyond antero-lingual border of M1.	Lingual margin of tooth sub-angular. Ratio of anterior to posterior width relatively low, tooth quadrangular in shape. Hypocone low but distinct, broad trough between hypocone and protocone. Lingual cingulum shallowly curved, postero-lingual margin more or less in line with antero-lingual border of M1.	Lingual margin of tooth shallowly curved. Ratio of anterior to posterior width slightly greater than that of *B. quadraticauda*, tooth sub-quadrangular in shape. Hypocone low but distinct; broad trough between hypocone and protocone; cingulum from hypocone to posterior short and shallowly curved; postero-lingual margin projects slightly beyond antero-lingual border of M1.
Number cuspids on i1 posterior to principal cusp in unworn dentition	Bicuspid.	Tricuspid.	Tricuspid, one specimen bicuspid.
Talonid of m1 and m2 (Fig. 5)	Talonid complete: low distinct mesoconid with oblique crest to hypoconid; low distinct hypoconulid; separate, distinct entoconid with a very low indistinct entoconid crest, scarcely linking to the metaconid.	Talonid with indistinct mesoconid as oblique crest to hypoconid, low hypoconulid, low but distinct entoconid linked by entoconid crest to metaconid. Entoconid in usually more evident on m1 than m2.	Talonid reduced: low mesoconid with low oblique crest to hypoconid, low but distinct hypoconulid but entoconid absent with a low indistinct trace of entoconid crest.
Talonid of m3 (Fig. 5)	Talonid incomplete: small but distinct mesoconid with oblique crest to hypoconid.	Talonid incomplete: elements comprise oblique crest to hypoconid.	Talonid incomplete: trace of mesoconid as oblique crest to low hypoconid.
Position of Foramen Ovale on Inferior Articular Facet (Fig. 4)	Central. Opens onto the inferior articular facet with a shallow depression towards the anterior.	Anterior. Opens anteriorly into the orbital region; antero-lateral roof formed by the pterygoid.	Anterior. Opens anteriorly into the orbital region; antero-lateral roof formed by the pterygoid.
Small foramen on rostrum anterior to infraorbital canal	Above P4, posterior to rostral fossa, within depression leading to infraorbital canal. One specimen with an additional foramen in the antorbital fossa above the junction of Un2 and Un3.	In rostral fossa above junction of P4 and Un4.	In rostral fossa above junction of P4 and Un4.
Extent of reticulation area of the wall of the mesopterygoid fossa	Extends to the base of the mesopterygoid fossa at the level of the hamular processes of the pterygoids and extends posteriorly well beyond hamular processes and close to the level of the vidian foramina.	Area of reticulation smaller than in *Parablarinella*, not extending to the base of the mesopterygoid fossa, barely posterior to hamular processes and far short of the vidian foramina.	Area of reticulation not extending to the base of the mesopterygoid fossa, nor extending posteriorly far beyond the level of the hamular processes and far short of the vidian foramina
Mandibular Foramen (MF) opens posteriorly leads anteriorly into the mandibular corpus and is ventral to the Ramal Foramen (RF) which opens dorsally into the postero-internal ramal fossa (or temporal fossa) (Fig. 6)	Mandibular foramen well separated from ramal foramen and clearly visible in lingual view. Ramal foramen posterodorsally positioned, largely concealed within the ventral border of the temporal fossa, not or barely visible in lingual view.	Mandibular foramen and ramal foramen occupy a shared fossa. Mandibular foramen not or barely visible in lingual view. Ramal foramen large and clearly visible in lateral view.	Mandibular foramen and ramal foramen in shared fossa but well separated. Ramal foramen small, posterodorsally positioned and visible in lingual view.
Coronoid spicule on buccal face of coronoid process	Prominent, projects posteriorly.	Moderately prominent not projecting far posteriorly.	Stout, not very prominent.

### Karyotype structure

The karyotyped specimens were assigned to *B. quadraticauda* and “*B. griselda*” (*Parablarinella*) based on the combination of molecular and morphological traits.

**Figure 7.**
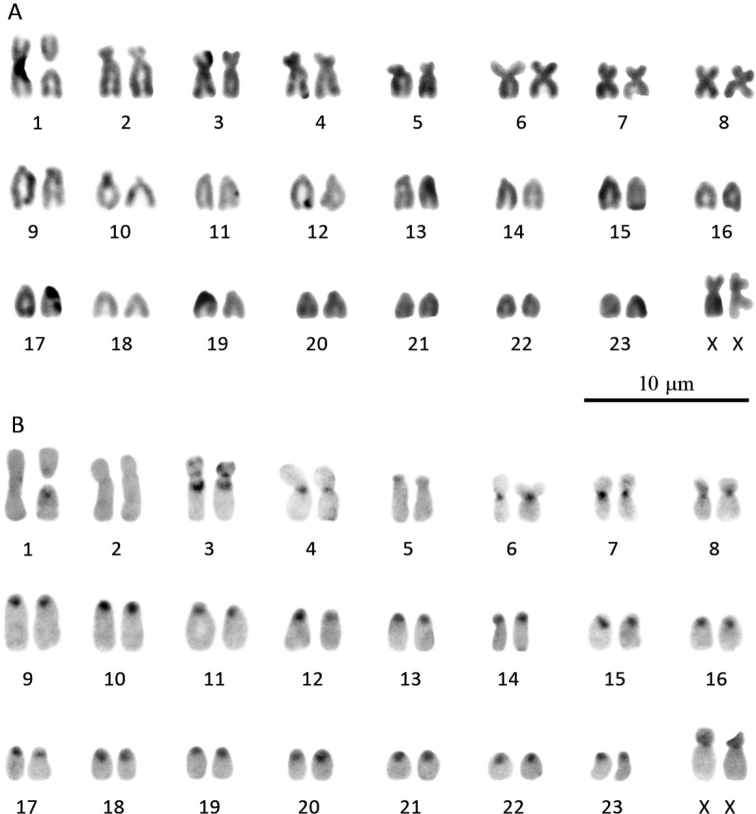
The female karyotype of *Blarinella quadraticauda* (G17-12) with 2*n* = 49, NF*a* = 62: routine Giemsa staining (**A**) and CBG-banding (**B**).

***Blarinella quadraticauda*.** The diploid chromosome number of the studied female (G17-12) was 2*n* = 49, and the fundamental autosome number (NF*a*) was 62 (Fig. 7A). The autosomal complement was represented by the largest polymorphic metacentric pair (# 1), three pairs of large submetacentrics (# 2–4), one medium-sized (#5) submetacentric pair, three medium-sized (#6–8) metacentric pairs, and 15 pairs of medium-sized to small acrocentrics (#9–23). The X chromosomes were medium-sized submetacentrics.

C-heterochromatic blocks (Fig. 7B) were revealed in the pericentric regions of all acrocentric autosomes (#9–23) as well as in four pairs of bi-armed autosomes (#4 and 6–8). Several C-blocks found in one submetacentric pair (#3) were localized interstitially and at telomeric region of the short arm. The polymorphic pair (#1) shown C-positive blocks in pericentric regions of both acrocentrics while the homologous metacentric was C-negative. Two pairs of medium-sized submetacentrics (#2 and 5) were C-negative. The X chromosomes had C-positive short arms.

**Figure 8.**
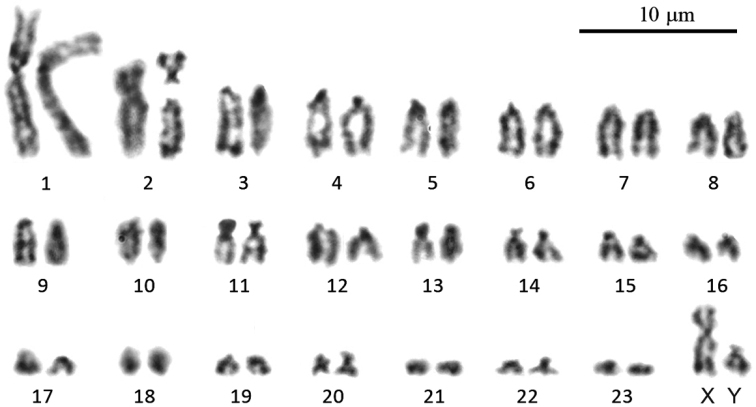
The male karyotype of *Parablarinella griselda* (ZMMU S-195179) with 2*n* = 49; NF*a* = 50.

***Parablarinella griselda*.** A short description of chromosome set of this male (2*n* = 49; NF*a* = 50; ZMMU S-195179) was previously reported in Sheftel et al. (2018) under the name Blarinella cf. griselda. Here we describe this karyotype in more details and present a karyogram of this specimen for the first time (Fig. 8). The studied male (ZMMU S-195179) had 2*n* = 49; NF*a* = 50. The autosomal part of the karyotype consisted of one largest metacentric pair (#1), one large polymorphic submetacentric pair (#2) and 21 pairs of medium-sized to small acrocentrics (#3–23). The X chromosome was the medium-sized submetacentric and the Y was a small acrocentric. Only conventional Giemsa staining was applied for this specimen because of a poor quality of chromosome suspension.

## Discussion

### Systematics and nomenclature

There is no doubt from the results of the current study that the clade comprising the holotype of *Blarinella griselda* and a few other specimens from southern Gansu, northern Sichuan and Shaanxi is the true *griselda* clade, which is highly divergent from the *B. quadraticauda* + *B. wardi* clade. We believe that *griselda* should be attributed to a separate genus because the age of divergence corresponds to that among recognized genera in the Soricinae (Dubey et al. 2007; He et al. 2010; our data). Recently, He et al. (2018) suggested the same taxonomic decision. However, without including comparisons with the holotype or topotypical material they had insufficient evidence for their attribution of the new name to *B. griselda* Thomas, 1912, and unfortunately the name *Pantherina* that they chose is already preoccupied by the same name for a subgenus of African beetles (Agrilus (Pantherina) Curletti, 1998). Thus, we provide a new replacement name for this genus: *Parablarinella* nom. nov. While some of the specific morphological traits of the new genus are highlighted in the description by He et al. (2018), a more detailed account is warranted and an enhanced diagnosis of the new genus is provided.

#### Family Soricidae Fischer, 1817

##### Subfamily Soricinae Fischer, 1817

###### Tribe Blarinini Stirton, 1930

####### Parablarinella nom. nov.

Taxon classificationAnimaliaSoricomorphaSoricidae

C63F24D9-4C6F-5910-9520-A9641EB26435

Pantherina He in He et al. 2018, not Pantherina Curletti, 1998 (Coleoptera).

**Type species.***Blarinella griselda* Thomas, 1912.

**Distribution.** Endemic to China. Known from a few specimens from southern Gansu, north-western Sichuan and southern Shaanxi.

**Etymology.** The name of the new genus is derived from the Greek word παρά “para” (near) and the generic name *Blarinella* previously attributed to this taxon. Gender is feminine.

**Amplified diagnosis.** A medium-sized shrew, externally similar in appearance to *Blarinella*. Genetically and karyotypically distinct from that genus and distinguished by a combination of the following craniodental characters. Angle of principal to posterior cusp of I1 moderately shallow, greater than 90°; Un3 smaller than Un2 but not markedly so; lingual margin of P4 curved, hypocone absent; talonid of m1 and m2 with a low distinct mesoconid and a separate, distinct entoconid with a very low indistinct entoconid crest scarcely linking to the metaconid; talonid of m3 with a small but distinct mesoconid. The foramen ovale is centrally positioned on the inferior articular facet; small foramen present on rostrum above P4, posterior to the rostral fossa, within depression leading to the infraorbital canal; reticulation of the wall of the mesopterygoid fossa extends to the base of the fossa and posteriorly beyond the hamular processes and close to the level of the vidian foramina; mandibular foramen well separated from ramal foramen and clearly visible in lingual view; ramal foramen posterodorsally positioned, largely concealed within the ventral border of the temporal fossa, not or barely visible in lingual view.

#### Comparison of karyotypes

Up to date, only three different karyotypes of Asiatic short-tailed shrews have been known – *Blarinella wardi* with 2*n* = 32, NF*a* = 58 (Moribe et al. 2007), Blarinella cf. quadraticauda (authors named this specimen *B. griselda*) with 2*n* = 44, NF*a* = 56 (Ye et al. 2006), and *Parablarinella griselda* (specimen ZMMU S-195179) karyotype (2*n* = 49; NF*a* = 50) described by Sheftel et al. (2018) under the name Blarinella cf. griselda without a karyogram. In this study we present for the first time the fourth karyotypic variant found among Asiatic short-tailed shrews species and belonging to *Blarinella quadraticauda*, as well as the karyogram of the specimen ZMMU S-195179 of *P. griselda*. Both studied specimens have an odd number of chromosomes because of a polymorphism of one of the autosomes, the first largest metacentric pair in the case of *B. quadraticauda* and the second bi-armed submetacentric pair in *P. griselda*. In spite of the same number of chromosomes (2*n* = 49), the karyotype structure of these two individuals differs substantially from each other. The *B. quadraticauda* karyotype contains eight pairs of bi-armed and 15 pairs of single-armed (acrocentric) chromosomes while *P. griselda* has only two pairs of bi-armed chromosomes and 21 pairs of acrocentrics. However, there is little difference in karyotype structure between *B. quadraticauda* from Gansu studied here and the specimen from Yunnan described in Ye et al. (2006). The latter has the same number of bi-armed (eight pairs) and 13 pairs of acrocentric chromosomes. Unfortunately, we were not able to apply GTG-banding for the studied specimen to determine the type of chromosomal rearrangements.

The karyotype of *B. wardi* has the lowest number of chromosomes (2*n* = 32) among all the Asiatic short-tailed shrews examined. The autosomal complement consists only of bi-armed chromosomes excluding the one single-armed pair of the smallest acrocentrics. Up to now there is no data about any differential staining for this species which could allow one to reveal structural rearrangements contributing to the karyotype divergence in this group. We conclude that the three species of Asiatic short-tailed shrews (*P. griselda*, *B. quadraticauda*, and *B. wardi*) demonstrate quite different karyotypic structure and chromosome morphology.

#### Distribution of Asiatic short-tailed shrews

The available genetic data suggest that most of the specimens of Asiatic short-tailed shrews from China and Vietnam previously recorded as *B. griselda* (Jiang et al. 2003; Lunde et al. 2003; Abramov et al. 2007; Hoffmann and Lunde 2008; Chen et al. 2012) and B. cf. quadraticauda (He et al. 2018) belong to the widespread and polymorphic species *B. quadraticauda*. This species may be sympatric with *B. wardi* in western Yunnan and northern Myanmar (Chen et al. 2012; Bannikova et al. 2017).

Our research not only proved the conspecificity of the holotype of *B. griselda* with specimens from southern Gansu and Sichuan, but also demonstrated that true *griselda* is more widespread than was suggested by He et al. (2018). Only two specimens of Asiatic short-tailed shrews were known from Gansu Province before this study. These include the holotype of *B. griselda* (NHMUK 1912.8.5.23, skull, skin) and specimen AMNH M-60449 (skin only, skull lost). The former was collected from “42 miles S.E. of Tao-chou, Tsin-ling Mountains, Kansu, 10000 feet” [68 km S.E. of Lintan, Qinling Mountains, Gansu, 3048 m] (in Lintan County, Gannan Prefecture) (Fig. 1, loc. 1), the latter has no exact locality, just “Kansu”.

Bannikova et al. (2017) listed the specimen of *Parablarinella* (ZMMU Chi-111) from the Taizishan National Reserve in southern Gansu, which on current knowledge appears to be the northernmost point of the range of *Parablarinella*. He et al. (2018) listed three specimens of the new genus from an unspecified locality in Qinling Mountains, southern Shaanxi (Fig. 1, loc. 5) where it is sympatrically distributed with “B. cf. quadraticauda”. The Qinling Mountains are an extensive mountain range, extending from Gansu in the west, the site of the type locality of *P. griselda*, to Shaanxi in the east, the location of the specimens recorded by He et al. (2018). The authors also noted that this species was not found in Chongqing, Hubei, or northwestern Sichuan. Recently however, this species was collected in two localities in northwestern Sichuan (G17-87, Zoigê and G18-252, Songpan; Fig. 1). As in Shaanxi, *P. griselda* may also occur here with *B. quadraticauda*; however, no data support this to date. It is possible that the two species prefer different elevational zones. All our specimens of *P. griselda* were collected in conifer and mixed forests at an altitude of 2800–3400 m, where they were trapped in the riparian growth along streams, while B. cf. quadraticauda (at one of its northernmost points in south-eastern Gansu, Huixian County, specimen G17-12) was found in the broadleaf (subtropical) forest at an altitude of ~1500 m. In Vietnam, this species is reported to occur in bamboo forests between 1500 and 1700 m elevation. However, typical *B. quadraticauda* from western Sichuan is also known to inhabit mountain conifer forests and the alpine zone (Hoffmann and Lunde 2008), thus, suggesting potential ecological plasticity of this species.

#### Phylogenetic position of *Parablarinella*

The molecular data clearly indicate that *Parablarinella* and *Blarinella* are phylogenetically close to *Blarina* and *Cryptotis* (Ohdachi et al. 2006; Dubey et al. 2007; He et al. 2018; this study). Although Thomas (1911) emphasized the similarity between *Blarina* and *Blarinella*, this view was discounted by Allen (1938) who considered that *Blarinella* was more closely related to *Sorex*. In his meticulous study of fossil Soricidae, Repenning (1967) divided the Soricinae into three tribes: the Soricini, to which *Blarinella* was assigned; the Blarinini, to which *Blarina* and *Cryptotis* were assigned; and the Neomyinae. The mandibular condyle with a broad interarticular area occurs in both the Soricini and Blarinini (as opposed to the narrow interarticular area in the Neomyini) but the Soricini (as in the Neomyini) were defined by the presence of an entoconid crest on the first two lower molars (m1 and m2), whereas the entoconid crest is absent in the Blarinini. In his classification of fossil and Recent shrews Reumer (1998), erected a separate tribe Blarinellini, for *Blarinella* and eight North American and Eurasian fossil genera, which was diagnosed by a combination of characters to separate this tribe from the other six tribes that he recognised. When the characters for the tribes Blarinellini and Blarinini are compared as in Table 4 it may be seen that the only substantive character to distinguish recent genera belonging to the two tribes is that in Blarinellini the entoconid on m1 and m2 is close to the metaconid and an entoconid crest is present, whereas in Blarinini the entoconid is separate from the metaconid and the entoconid crest is absent. However, based on molecular data, Dubey et al. (2007) suggested that *Blarinella* should be allocated to Blarinini. We concur with this view taking into account the level of genetic divergence between *Blarinella* and *Blarina* recovered in our study. It remains to be established which morphological characters should be regarded as synapomorphies for Blarinini in the wider sense. It is noteworthy that *Parablarinella* is characterized by a prominent entoconid with an indistinct entoconid crest, which is a condition considered to be synapomorphy of Blarinini by Reumer (1998), while in *B. wardi* both the entoconid and entoconid crest are reduced. Consequently, *Blarinella* and *Parablarinella* should be considered as members of the tribe Blarinini Stirton, 1930 instead of Blarinellini Reumer, 1998. In this respect, the molecular results are consistent with the fossil data, which reveal parallelisms in reduction of the entoconid crest in several fossil genera attributed to Blarinini and Blarinellini (Doby 2015). However, a detailed analysis of relationships among Neogene lineages deserves a separate study.

**Table 4.** Compilation of characters used by Reumer (1998) for definition of the tribes Blarinellini and Blarinini. Distinctive characters are indicated by bold typeface.

**Blarinellini (*Blarinella, Parablarinella*)**	**Blarinini (*Blarina*, *Cryptotis*)**
Horizontal ramus of mandible short and high, making the lower dentition compressed anteroposteriorly and giving the lophs and lophids a compressed W-shaped appearance^#^	Lower molars W-shaped*
Mandible with a broad interarticular area^#^	Mandible with a broad interarticular area^#^
Mandibular condyle with its articular facets separated*	Mandibular condyle with its articular facets separated^#^
Coronoid spicule well developed^#^	Coronoid spicule present*
Internal temporal fossa of moderate size*	Internal temporal fossa of moderate size^#^
**Lower molars with the entoconid close to the metaconid so that the entoconid crest is short and high** (N.B. specific variation)	Lower molars with the entoconid separate from the metaconid and* lacking the entoconid crest
M3 with a reduced talonid^#^	M3 with a reduced talonid*
Teeth heavily pigmented^#^	Pigmented^#^
Upper incisor protruding but not fissident^#^	Upper incisor not fissident^#^
Upper molariform teeth with a reduced posterior emargination, showing a tendency to develop a continuous endoloph^#^	Slight emargination*
Occlusal surface of M1 nearly square^#^	Variable sub-square or oblong*

^#^ Characters specified by Reumer (1998) * Character state not mentioned in Reumer (1998) but observed in specimens of *Blarinella, Parablarinella, Blarina* and *Cryptotis* in this study

## Supplementary Material

XML Treatment for Parablarinella

## Supplementary materials

Supplementary material 1 Figures S1, S2; Tables S1–S3 Explanation note: **Figure S1.** The phylogenetic relationships in *Blarinella* as reconstructed in MrBayes based on the extended alignment of *cytb*. **Figure S2.** The phylogenetic relationships in *Blarinella* as reconstructed in MrBayes based on the alignment of *ApoB*. **Table S1.** GenBank accession numbers of sequences retrieved from GenBank and newly collected sequences used in the study (marked in bold). **Table S2.** Primers for *cytb* amplification and sequencing. **Table S3.** The best-fit substitution models employed for each of the five partitions found by IQTREE. https://binary.pensoft.net/file/355143This dataset is made available under the Open Database License (http://opendatacommons.org/licenses/odbl/1.0/). The Open Database License (ODbL) is a license agreement intended to allow users to freely share, modify, and use this Dataset while maintaining this same freedom for others, provided that the original source and author(s) are credited.Anna A. Bannikova, Paulina D. Jenkins, Evgeniya N. Solovyeva, Svetlana V. Pavlova, Tatiana B. Demidova, Sergey A. Simanovsky, Boris I. Sheftel, Vladimir S. Lebedev, Yun Fang, Love Dalen, Alexei V. Abramov
