# Supplementary material for: Who are you, Griselda? A replacement name for a new genus of the Asiatic short-tailed shrews (Mammalia, Eulipotyphla, Soricidae): molecular and morphological analyses with the discussion of tribal affinities
Source: Zookeys. 2019 Nov 11;888:133–58. doi: 10.3897/zookeys.888.37982 (PMC6861342; doi:10.3897/zookeys.888.37982)
Supplement: Supplementary material 1 [file zookeys-888-133-s001.pdf]

Table S1. Sequences retrieved from GeneBank. The voucher number or collection code is presented for the nuclear sequences of *Blarinella* spp. according to Fig. 4 and literature source.

species	GeneBank Acc No	Source
	cytb	
Blarinella wardi	JF719714	Chen et. al. 2012
B. quadraticauda	JF719701–F719713, JF719715–JF719725	Chen et. al. 2012
	NC_023950	Wang et al., 2014
	GU981258, GU981259	He et al., 2010
	AB175144	Ohdachi et al., 2006
Cryptotis parva	AB175135	Ohdachi et al., 2006
	ApoB	
B. quadraticauda	JF719697 (ElsmypA02001)	Chen et al., 2012
	DQ630187 (BLG)	Dubey et al., 2007
	LC124841 (AMNH101610)	Sato et al., 2016
	GU981108 (Blagris1), GU981109 (Blagris2)	He et al., 2010
Blarinella wardi	JF719698	Chen et al., 2012
Crocidura fuliginosa	GU981117	He et al., 2010
Pseudosoriculus fumidus	GU981121	
Soriculus nigrescens	GU981132	
Nectogale elegans	GU981128	
Episoriculus leucops	GU981122	
Chimarrogale himalayica	GU981112	
Chodsigoa hypsibia	GU981110	
Episoriculus macrurus	GU981125_	
Episoriculus caudatus	GU981119	
Chodsigoa parca	KX765554	Chen et al., 2017
Chodsigoa sodalis	DQ630191	Dubey et al., 2007
	BRCA1	
Blarinella quadraticauda	DQ630268 (BLG)	Dubey et al., 2007
	LC124932 (AMNH101610)	Sato et al., 2016
	GU981183 (Blagris1), GU981184 (Blagris2)	He et al., 2010
Cryptotis parva	DQ630261	Dubey et al., 2007
Crocidura fuliginosa	GU981192	He et al., 2010
Episoriculus macrurus	GU981202	
Episoriculus leucops	GU981197	
Nectogale elegans	GU981203	
Soriculus nigrescens	GU981209	
Episoriculus caudatus	GU981195	
Pseudosoriculus fumidus	DQ630277	
Chodsigoa parca	GU981189	
Chodsigoa hypsibia	KX765637	Chen et al., 2017
Chodsigoa sodalis	DQ630271	Dubey et al., 2007
Chimarrogale himalayica	DQ630260	
	RAG2	
Blarinella quadraticauda	GU981441 (Blagris1)	He et al., 2010
Crocidura fuliginosa	GU981450	
Pseudosoriculus fumidus	GU981454	

Soriculus nigrescens	GU981467	
Nectogale_elegans	GU981461	
Episoriculus_macrurus	GU981458	
Episoriculus leucops	GU981455	
Episoriculus caudatus	GU981452	
Chodsigoa parca	GU981446	
Chodsigoa hypsibia	GU981443	
Chodsigoa sodalis	GU981449	
Chimarrogale himalayica	KY082637	Abramov et al., 2017

Abramov AV, Bannikova AA, Lebedev VS, Rozhnov VV (2017b) Revision of Chimarrogale (Lipotyphla: Soricidae) from Vietnam with comments on taxonomy and biogeography of Asiatic water shrews. Zootaxa 4232: 216–230. https://doi.org/10.11646/zootaxa.4232.2.5

Chen S, Liu S, Liu Y, He K, Chen W, Zhang X, Fan Z, Tu F, Jia X, Yue B (2012) Molecular phylogeny of Asiatic short-tailed shrews, genus *Blarinella* Thomas, 1911 (Mammalia: Soricomorpha: Soricidae) and its taxonomic implications. Zootaxa 3250: 43–53.

Chen ZZ, He K, Huang C, Wan T, Lin LK, Liu SY, Jiang XL (2017) Integrative systematic analyses of the genus *Chodsigoa* (Mammalia: Eulipotyphla: Soricidae), with descriptions of new species Zoological Journal of Linnaean Socity 180: 694–713. https://doi.org/10.1093/zoolinnean/zlw017.

Dubey S, Salamin N, Ohdachi SD, Barriere P, Vogel P (2007) Molecular phylogenetics of shrews (Mammalia: Soricidae) reveal timing of transcontinental colonizations Molecular Phylogenetics and Evolution 44: 126–137. https://doi.org/10.1016/j.ympev.2006.12.002.

He K, Li Y-J, Brandley MC, Lin L-K, Wang Y-X, Zhang Y-P, Jiang X-L (2010) A multi-locus phylogeny of Nectogalini shrews and influences of the paleoclimate on speciation and evolution. Molecular Phylogenetics and Evolution 56: 734–746. doi.org/10.1016/j.ympev.2010.03.039

Ohdachi SD, Hasegawa M, Iwasa MA, Vogel P, Oshida T, Lin LK, Abe H (2006) Molecular phylogenetics of soricid shrews (Mammalia) based on mitochondrial cytochrome b gene sequences: with special reference to the Soricinae. Journal of Zoology 270: 177–191. https://doi.org/10.1111/j.1469-7998.2006.00125.x

Sato JJ, Ohdachi SD, Echenique-Diaz LM, Borroto-Paez, R, Begue-Quiala G, Delgado-Labanino JL, Gamez-Diez J, Alvarez-Lemus J, Nguyen ST, Yamaguchi N, Kita M (2016) Molecular phylogenetic analysis of nuclear genes suggests a Cenozoic over-water dispersal origin for the Cuban solenodon. Scientific reports 6: 3117.

Wang Q, Fu C, Chen S, Yong B, Chen G, Hao Z (2014) The complete mitogenome of Asiatic Short-tailed Shrew *Blarinella quadraticauda* (Soricidae). Mitochondrial DNA 27: 282–283. https://doi.org/10.3109/19401736.2014.892078.

Table S2. The original primers designed for amplification and sequencing of *cytb* in *Blarinella.*

№	primer	sequence (5`-3`)
		Blarinella spp.
external primers for complete *cytb* gene (1140 bp)		
1.	L14132_B	GACCTATGACATGAAAAATCATCGTTGT
2.	L14734_B	CATGAAAAATCATCGTTGTTATTCAACT
3.	H15327_B	GGTTTACAAGACCAGTGTAATAATTATACTAC
4.	H15347_B	GAATCTCCATTTCTGGTTTACAAGAC
5.	H15416_B	AGAATTCCAGCTTTGGGTGTTG
Internal primers for fragment 1-670 bp		
7	H400a	GCCTCAAAAAGACATTTGTCCTCAT
8	L240a	CTACAYGCCAACGGAGCATCAATA
9	H330a	AATAATACCCCGATRTTTCATGTTTCTA
10	L170ax	GACACAATAACAGCCTTTTCATCAGTAAC
11	H190a	ATCYCGGCARATGTGTGTTACTGATGA
12	L62a	TACCYGCTCCATCTAAYATTTCATCAT
13	H95a	GCAGACACCTAATAAAGAACCGAAGT
14	L360a	TTCGCRGTTATAGCYACAGCCTTTATA
15	H511a	AAYCGTGTGAGTGTTGCTTTGTC
16	L467a	TTGGCTCRGACCTWGTMCAATGAATCT
17	L580a	GAGTTCACCTATTATTCCTCCACGA
18	H601a	GGGTTGTTGGAACCAGTTTCGTG
19	H716a	TCAGGGGAGAATAATACTAATGATGTTA
20	H670a	GGAGAACACCTAGAATATCTTTRATTGTATA
		Blarinella griselda
Internal primers for fragment 1-670 bp		
21.	H670x	AGTGCTCCTAGAATGTCTTTGATTGTATA
22.	H400x	GCGCCTCAAAATGATATTTGTCCTC
23.	L240x	ATACATGCAAATGGAGCATCAATA
24.	H330x	AGCAGTACTCCGATATTTCATGTTTCTA
25.	H601x	GATGGATTGTTTGATCCTGTTTCGTG
26.	H190x	CTCGGCAAATGTGTGTTACTGATGA
27.	L62x	TACCTGCTCCATCCAAYATTTCATCAT
28.	H95x	GCAGATTCCTAATAGTGARCCGAAGT
29.	L360x	TTCGCAGTTATAGCYACTGCCTTTATA
30.	H511x	AACCGTGTGAGTGTTGCTTTGTC

31.	L467x	TTGGCTCAGACCTAGTAGAATGAATCT
32.	L580x	GGAGTCCACCTCTTATTTCTACACGA

Table S3. GenBank AccNos of the original sequences used in the study.

species	ID	voucher	*cytb*	*ApoB*	*IRBP*	*RAG2*	*BRCA1*
B. quadraticauda	36 (Bl-1)	ZIN 91211	*KY249525*	–	–	–	–
B. quadraticauda	42 (Bl-2)	ZIN 96272	–	*KY249531*	////////	*KY249542*	////////
B. quadraticauda	43 (Bl-3)	ZIN 96273	*KY249526*	–	–	*KY249543*	–
B. quadraticauda	136 (Bl-5)	ZIN 97788	*KY249527*	*KY249532*	–	*ZIN 97788*	////////
B. quadraticauda	V12-40	ZIN 101574	*KY249528*	*KY249533*	*KY249551*	*KY249545*	////////
B. quadraticauda	V12-61	ZIN 101575	*KY249529*	*KY249534*	*KY249552*	*KY249546*	////////
B. quadraticauda	G17-12	ZMMU G17-12	////////	////////	////////	////////	////////
Parablarinella griselda	Chi111	ZMMU 195179	*KY249530*	*KY249535*	////////	*KY249547*	////////
P. griselda	G17-87	ZMMU G17-87	////////	////////	////////	////////	–
P. griselda	G18-252	ZMMU G18-252	////////	////////	////////	////////	–
P. griselda		NHMUK 1912.8.5.23	////////				–
Chodsigoa parca	AVA10-29	ZIN99787	–	*KY082620*	–	*KY082638*	–
Chodsigoa hypsibia	Chi11-72	ZMMU 195190	////////	*MF577031*	*KY082659*	*KY082640*	////////
Chodsigoa hypsibia	G17-13	ZMMU G17-13	////////	–	–	–	–

sequences used from our previous study are marked in *Italic*

Table S4. The best-fit substitution models employed for each of the five partitions found by IQTREE.

model	Partitions (gene and codon position)
TIM3+F+G4	*ApoB* 1st and 2d codon positions; *IRBP* 2d codon position
HKY+F+G4	*BRCA1* and IRBP 1st codon position
K2P+I	*RAG2* 1st and 2d codon positions
TPM2+F	*IRBP* 3rd codon position
K3Pu+F+G4	*BRCA1* 2d codon position; *ApoB*, *BRCA1*, *RAG2* 3rd codon positions

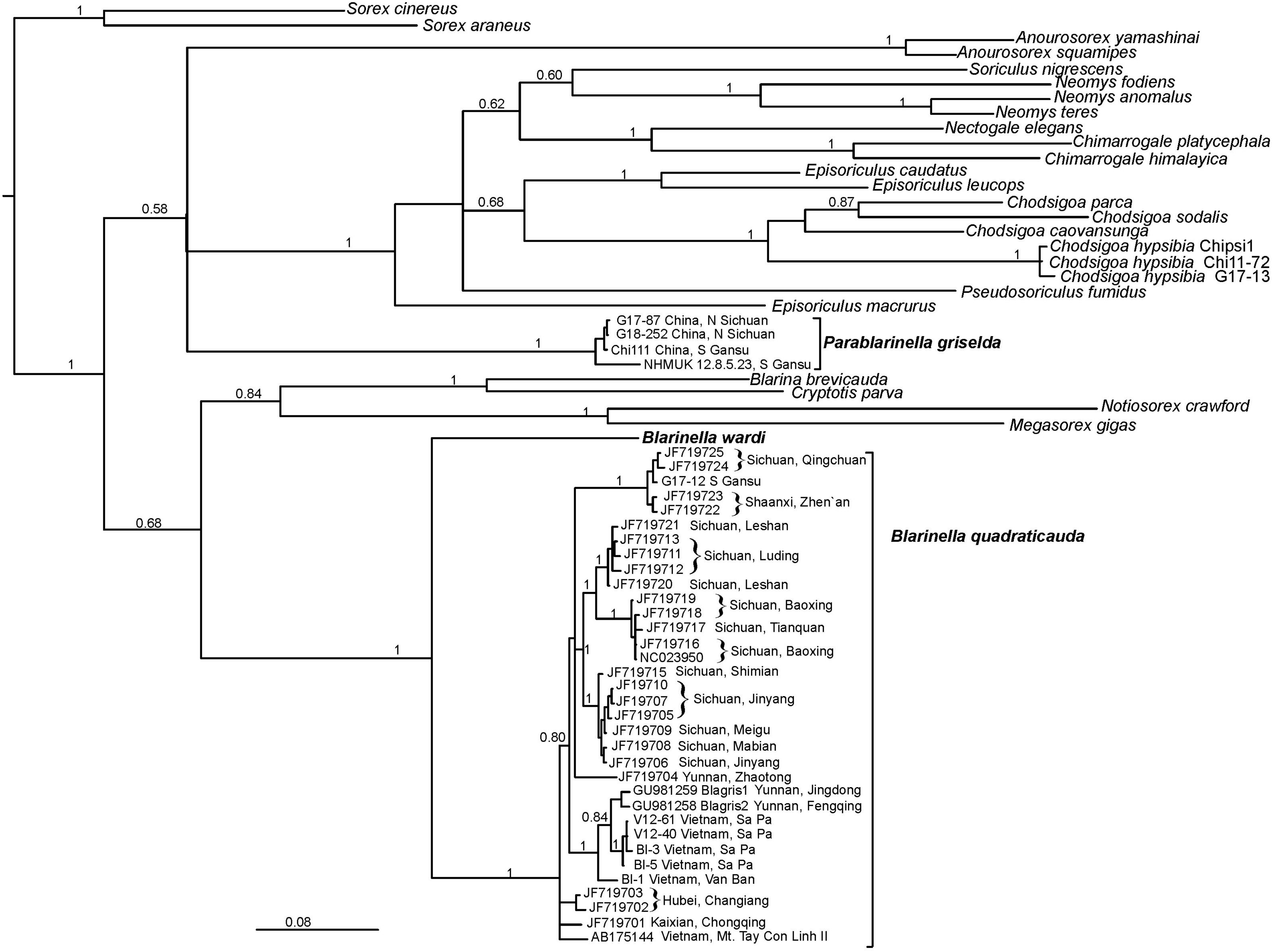
Sorex cinereus
Sorex araneus
Anourosorex yamashinai
Anourosorex squamipes
Soriculus nigrescens
Neomys fodiens
Neomys anomalus
Neomys teres
Nectogale elegans
Chimarrogale platycephala
Chimarrogale himalayica
Episoriculus caudatus
Episoriculus leucops
Chodsigoa parca
Chodsigoa sodalis
Chodsigoa caovansunga
Chodsigoa hypsibia Chipsi1
Chodsigoa hypsibia Chi11-72
Chodsigoa hypsibia G17-13
Pseudosoriculus fumidus
Episoriculus macrurus
G17-87 China, N Sichuan
G18-252 China, N Sichuan
Chi111 China, S Gansu
NHMUK 12.8.5.23, S Gansu
Parablarinella griselda
Blarina brevicauda
Cryptotis parva
Notiosorex crawford
Megasorex gigas
Blarinella wardi
JF719725
JF719724
Sichuan, Qingchuan
G17-12 S Gansu
JF719723
JF719722
Shaanxi, Zhen`an
Blarinella quadraticauda
JF719721 Sichuan, Leshan
JF719713
JF719711
JF719712
Sichuan, Luding
JF719720 Sichuan, Leshan
JF719719
JF719718
Sichuan, Baoxing
JF719717 Sichuan, Tianquan
JF719716
NC023950
Sichuan, Baoxing
JF719715 Sichuan, Shimian
JF19710
JF19707
JF719705
Sichuan, Jinyang
JF719709 Sichuan, Meigu
JF719708 Sichuan, Mabian
JF719706 Sichuan, Jinyang
JF719704 Yunnan, Zhaotong
GU981259 Blagris1 Yunnan, Jingdong
GU981258 Blagris2 Yunnan, Fengqing
V12-61 Vietnam, Sa Pa
V12-40 Vietnam, Sa Pa
Bl-3 Vietnam, Sa Pa
Bl-5 Vietnam, Sa Pa
Bl-1 Vietnam, Van Ban
JF719703
JF719702
Hubei, Changiang
JF719701 Kaixian, Chongqing
AB175144 Vietnam, Mt. Tay Con Linh II
0.08
1
0.58
0.60
0.62
0.68
0.87
0.84
0.80
